# Improving Management of Green Retrofits from a Stakeholder Perspective: A Case Study in China

**DOI:** 10.3390/ijerph121113823

**Published:** 2015-10-28

**Authors:** Xin Liang, Geoffrey Qiping Shen, Li Guo

**Affiliations:** 1 Department of Building and Real Estate, Hong Kong Polytechnic University, Hong Kong, China; E-Mail: xin.c.liang@connect.polyu.hk; 2 School of Economics and Management Engineering, Beijing University of Civil Engineering and Architecture, Beijing 100044, China; E-Mail: guoli@burea.edu.cn

**Keywords:** Green retrofit, critical success factors, stakeholders, social network analysis

## Abstract

Green retrofits, which improve the environment and energy efficiency of buildings, are considered a potential solution for reducing energy consumption as well as improving human health and productivity. They represent some of the riskiest, most complex, and most uncertain projects to manage. As the foundation of project management, critical success factors (CSFs) have been emphasized by previous research. However, most studies identified and prioritized CSFs independently of stakeholders. This differs from the reality, where the success of green retrofits is tightly interrelated to the stakeholders of projects. To improve the analysis from a stakeholder perspective, the present study proposed an innovative method based on a two-mode social network analysis to integrate CSF analysis with stakeholders. The results of this method can provide further understanding of the interactions between stakeholders and CSFs, and the underlying relationship among CSFs through stakeholders. A pilot study was conducted to apply the proposed method and assess the CSFs for green retrofits in China. The five most significant CSFs are identified in the management of green retrofit. Furthermore, the interrelations between stakeholders and CSFs, coefficient and clusters of CSFs are likewise discussed.

## 1. Introduction

In 2010, China replaced the United States (U.S.) as the largest energy consumer by accounting for more than 20% of the total energy consumption in the world [1]. The gap between China and the U.S., which is the second-largest energy consumer, continues to widen because while the latter has reduced its energy consumption in the past years, the former has increased it by 10% on average since 2009 [2]. As a result of its high energy consumption, China is also the leading energy-related CO_2_ emitter in the world [2]. The building sector is responsible for almost half of the energy consumption [3] and greenhouse gas emissions [4]. Moreover, more than 80% of the life-cycle building energy consumption occurs during the actual occupancy operation stage, rather than the construction stage [5]. Therefore, the energy efficiency of existing buildings is a key issue related to the total energy consumption and greenhouse gas emissions.

Energy efficiency retrofits for existing buildings are considered an important method to achieve the targets of energy reduction and sustainable development. They can significantly benefit the environment, society, and economy by improving energy efficiency [6,7], reducing emissions [8,9], controlling resource usage [10], enhancing the reputation of building owners [11], improving the health and productivity of occupants [12,13], reducing operation costs [14], increasing rent and occupancy rates [15,16], and creating job opportunities [17].

Retrofit projects are considered “one of the most risky, complex, and uncertain within the construction industry” [18]. An energy efficiency retrofit project is more complex and riskier than a general retrofit project, as well as more difficult than constructing a new green building [19], because of the lack of existing building information [18], complicated cost sharing [19], and increased stakeholder interactions [13]. Therefore, to improve the energy efficiency retrofit projects, there is an urgent need to develop a set of critical success factors (CSFs) integrated with the relationship of stakeholders.

Stakeholders can be defined as a person or a group of persons, who are influenced by or able to influence a retrofit project [20]. The CSFs are the essentials for “manage success” [21]. Both CSFs and stakeholders have gained increasing attention from scholars [17,21]. However, most studies have focused on either CSFs or stakeholders, and only a few have analyzed CSFs integrated with stakeholders. In construction projects, stakeholders have been identified as important for accomplishing a successful project, which means stakeholders have strong correlations with CSFs. For example, the CSFs being able to “develop appropriate organization structure” may relate to the organization of the project team with clients, designers, and contractors, whereas the CSFs’ “policy support” is closely connected with the government. Therefore, previous research, which discussed CSFs independently, ignored the correlation between CSFs and stakeholders, as well as the internal relations among CSFs, through the links of stakeholders.

The current research aims to develop a set of CSFs for energy efficiency retrofit projects and the interrelation between CSFs and stakeholders. Social network analysis (SNA) and stakeholder analysis are integrated to evaluate the CSFs. The relationship between CSFs and stakeholders can be modeled by a two-mode network [22]. This paper first provides a critical review of the literature about energy efficiency retrofits, CSFs, and the main stakeholders involved in energy efficiency retrofit projects. Then, it introduces an innovative method that models and processes data based on the two-mode network. Based on this method, a workshop was conducted on some participants who have been involved in energy efficiency retrofit projects in China. Through this workshop, a matrix of CSFs and stakeholders was identified. Projection, a method of two-mode network analysis, was then used to investigate the underlying relationships among the identified CSFs. Moreover, graph theory-based analysis was used to establish the priority, cluster, and coefficient of these CSFs. The result of this study can help understand the underlying relationship between CSFs and stakeholders, as well as improve the management success in energy efficiency retrofit projects.

## 2. Literature Review

### 2.1. CSFs for Green Retrofit Management

Several studies have focused on the CSFs for energy efficiency retrofits in recent years. The use of CSFs is considered an effective method for achieving “management success” [21]. Studies have identified factors underpinning the success of project management in construction projects, such as analyzing the needs of stakeholders, managing risk, and planning the project [23]. Xu, Chan and Qian [17] proposed a set of CSFs for energy performance contracting (EPC) for the sustainable building energy efficiency retrofit of hotel buildings by using the factor analysis method. Yang and Zou [24] focused on the risk factors of green building projects by SNA.

The current literature usually classifies the CSFs of energy efficiency retrofit projects into different categories. Ma, Cooper, Daly and Ledo [16] identified CSFs as human factors, retrofit technologies, policies and regulations, client resources, building-specific information, and other uncertain factors. Mickaityte, *et al.* [25] analyzed the factors from other dimensions, namely, social, ecological, economic, cultural, architectural, and technical. Menassa and Baer [26] stated that energy efficiency retrofit should balance economic, environmental, and social factors from a sustainability perspective. Rey [6] classified factors into environmental, sociocultural, and economic fields.

Scholars have not reached a consensus on the standard classification of CSFs. For this study, CSFs are classified into five categories to help industry practitioners identify them for the proposed method. These categories are economics (related to economics, investment, finance, and tax), building information and environment (related to existing building information, evaluation, and environment), sociocultural (related to social, culture, and humanity), technology (related to technology and facilitated tools), and policy and standards (related to policy, standards, and program). It should be noted that this categorization does not cover all CSF groups. It only emphasizes that energy efficiency retrofit is different from general construction projects. Table 1 summarizes the CSFs for energy efficiency retrofit identified in the literature review.

**Table 1:** The CSFs of energy efficiency retrofit identified in previous literature.

Code	CSF	Literature
CSF1	Cost	[14,27,28]
CSF2	Who invest	[15,26]
CSF3	Profit distribution among stakeholders	[29]
CSF4	Interruptions in operations	[19,30]
CSF5	Interest rate	[27]
CSF6	Occupancy type	[16,31]
CSF7	Who get energy saving benefit	[15]
CSF8	Subsidies/tax reduction	[16,27,29]
CSF9	Rent increasing after retrofit	[8,15]
CSF10	Existing building environment	[16,25]
CSF11	Existing building condition	[17,18,25,26]
CSF12	Existing facilities condition	[25,26,32]
CSF13	Existing building information modeling (BIM)	[32,33]
CSF14	Existing building evaluation	[25]
CSF15	Clear vision	[11]
CSF16	Cooperation among stakeholders	[11,28]
CSF17	Information sharing	[16,25]
CSF18	Users’ behavior and demand analysis	[16,34]
CSF19	Project organization and management	[28]
CSF20	Experience sharing and education	[29,30,31]
CSF21	Cultural traditions	[25]
CSF22	Maturity of technology	[16,25]
CSF23	Complexity of technology	[29]
CSF24	Maintainability	[34]
CSF25	Information technologies and computerization level	[25]
CSF26	Collaborative design and automation	[12,18,35]
CSF27	Clear government programs and policies	[16,25,29]
CSF28	Clear criteria and standards	[17,19,25,31]

### 2.2. Main Stakeholders in Energy Efficiency Retrofits

To deeply mine the interactions of CSFs, the links between CSFs and stakeholders should be identified because all CSFs interact with one another through stakeholders. That is, stakeholders are the medium of the interactions among CSFs. Boecker, *et al.* [36] emphasized that the earlier all stakeholders engage in the design process, the more success can be achieved. However, the previous literature investigated CSFs and stakeholders as two independent systems without considering the links between them. The relationships between CSFs and stakeholders are difficult to determine for the following reasons: (1) some stakeholders potentially influence CSFs, which leads to unobvious relationships; (2) the stakeholders identified by previous studies were not comprehensive and systematic, and thus some stakeholders related to CSFs were not emphasized. For example, non-government organizations (NGOs) and communities may influence projects [37]. Moreover, tenants and facility managers are important in existing buildings but are rarely investigated [26]. Therefore, stakeholders should be studied systematically to identify their relationship with CSFs.

In energy efficiency retrofits, stakeholders are the people who directly or indirectly have a vested interest in the building, its operation, and the outcome of a future retrofit project [26]. Klotz and Horman [13] indicated that the successful delivery of sustainable projects generally involves stakeholder interactions. According to previous studies [12,16], the process of green retrofit projects can normally be divided into several phases, including pre-retrofit survey and planning, design, site implementation, validation and utilization. Berardi [38] indicated that different stakeholders have different initial involvement times. For example, owners are normally involved since the beginning, whereas designers and contractors join a project at the design and implementation phases, respectively. Furthermore, powers of stakeholders vary in different organizations and phases [38,39]. Since this study emphasizes on the method of two-mode social network analysis, to demonstrate how to apply this method, the project is considered as a whole rather than several phases in the case study. The stakeholders and their influence on project are analyzed synthetically. Based on this research, stakeholders in different phases can likewise be analyzed with this method. It is further discussed in Section 5.

The key stakeholders in energy efficiency retrofit are the clients, users, designers, contractors, suppliers, facility managers, local authorities, government, and institutions [27]. Ali, Rahmat and Hassan [18] specified that the key stakeholders are clients, architects, engineers, main contractors, quantity surveyors, and specialist contractors. Miller and Buys [19] emphasized that owners, managers, occupants, and contractors have the most influence on the cooperation in multi-tenant commercial buildings. Energy service companies have been the focus of some previous studies [17,19]. The main stakeholders are listed in Table 2.

**Table 2:** Main stakeholders of energy efficiency retrofit identified in previous literature.

Code	Stakeholder	Literature
S1	Owner/client	[19,27,28,40,41]
S2	Occupier/user	[19,27,41]
S3	Facilities manager	[19,27,41]
S4	Designer	[27,28,40,41]
S5	Contractor	[19,27,28,40]
S6	Supplier	[27,41]
S7	Sub-contractor	[18]
S8	Government	[27,41]
S9	Financial institution/bank	[19,41,42]
S10	Energy service company	[17,19]
S11	Industry institution	[27]
S12	NGO/community	[37,40]
S13	Research institution	[43,44]

The corresponding definitions of each stakeholder are as follows:

(1) Owner/client: Owns the existing building or the client of the retrofit project
(2) Occupier/user: Occupies the existing building
(3) Facility manager: Manages the facilities in the existing building
(4) Designer: Responsible for the people involved in the retrofit design, including architect, structural engineer, and electrical engineer
(5) Contractor: Responsible for the people involved in the retrofit construction, including project manager, purchasing manager, and quantity surveyor
(6) Supplier: Provides materials and machines
(7) Sub-contractor: Has a subcontract from the contractor; subcontracts mainly from a specific technology field, such as waste management, landscaping, roofing, painting, tiling, and fire services
(8) Government: Establishes policies, laws, and plans related to energy efficiency retrofit
(9) Financial institution/bank: Provides loans and financial support
(10) Energy service company: Includes the energy (i.e., power, water, and gas) provider and EPC servicer
(11) Industry institution: Has the authority to define the industrial standards and evaluate projects; examples are the Green Building Council and American Society of Civil Engineers
(12) NGO/community: Promotes sustainable development, low carbon emission, and energy efficiency
(13) Research institution: Includes universities and other research institutions

## 3. Methods

This study proposes a method based on the stakeholder-associated two-mode SNA to help evaluate the CSFs and their correlation with stakeholders. SNA has been used in various construction areas, but the application of two-mode social network theory to stakeholder- associated analysis of CSFs for energy efficiency retrofit remains unexplored.

**Figure 1:**
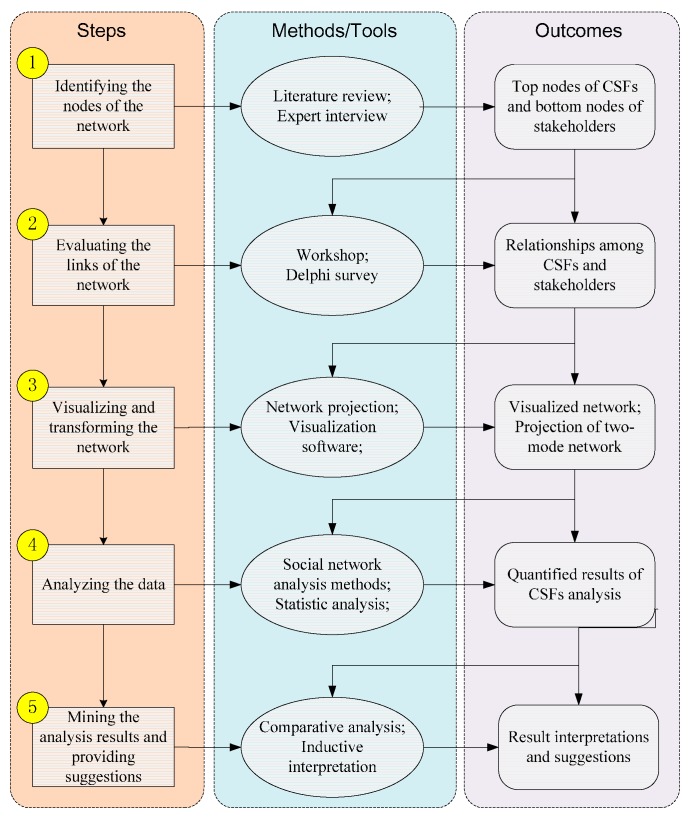
Framework of the CSFs evaluation method based on SNA.

The main steps of the proposed method are as follows: (1) identifying the nodes of the network, including top nodes of CSFs and bottom nodes of stakeholders; (2) evaluating the links of the network that indicate the relationships among nodes; (3) visualizing and transforming the network; (4) analyzing data; and (5) mining the meaning of the analysis results and providing suggestions. Figure 1 illustrates the proposed method.

### 3.1. Step 1: Identification of the Nodes in the Network

Networks comprise nodes and links. The first step is to identify the nodes in the network to develop an appropriate list of CSFs and stakeholders. Two groups of nodes are defined in this model, namely, CSFs and stakeholders. Several methods are used to facilitate this step, including literature review and experience-based methods (e.g., expert interviews, focus groups, semi-structured interviews, and survey). These methods can be applied synthetically according to different situations.

Literature reviews are an efficient method to identify CSFs and stakeholders. Related studies, surveys, and reports can serve as a reference. Although previous studies analyzed from different perspectives or areas, a relatively complete list can be obtained by combining their results together. This method is widely used in factor identification. For example, Xu, Chan and Qian [17] identified several CSFs of EPC for the sustainable building retrofit of hotel buildings. Yang, Shen, Drew and Ho [21] listed 15 CSFs for stakeholder management in construction projects through a literature review. The stakeholder requirements of sustainable retrofit were also obtained from the literature [26,45]. The advantages of the literature review method include: (1) efficient identification of factors, (2) reliable references for identified factors, and (3) analysis of the popularity and development trend of factors. However, the literature review is unable to find undefined factors. Therefore, it is suitable only for summarizing factors, rather than exploring them.

By contrast, experience-based methods are widely used in the identification of CSFs and stakeholders. Some stakeholders involved in related projects are invited to attend interviews or workshops to identify the key stakeholders and CSFs according to their practical experiences [24,46]. In terms of advantages, the experience-based methods use a relatively short time and make wise decisions for most conventional projects [24]. However, this method has difficulty listing the complete factors because it depends on the limited experience of interviewees or participants. Snowball sampling, which iteratively extends the involved participants, can relieve this limitation. Some new factors may be explored during the snowball sampling, and the resulting completeness is correlated with the iterations. Lienert, *et al.* [47] identified the stakeholders in water infrastructure planning, and Prell, *et al.* [48] identified stakeholders in nature resource management. Although this snowball sampling method could break cognitive limitations, it is time consuming and has some practical and ethical issues [24].

### 3.2. Step 2: Evaluation of the Links in the Network

This step aims to determine the links in a network, which represent the relationships among the nodes. The link value means the tightness degree between the stakeholder and CSFs. The link value is high when the stakeholder has a high influence on the CSF or the CSF affects the stakeholder significantly, and vice versa. The CSF-stakeholder evaluation matrix is shown in Table 3. Stakeholders are coded with S# and CSFs with CSF#, where # is the number of the respective stakeholder and CSF. The number on the cross-point indicates the tightness degree between the corresponding stakeholder and CSF. This paper uses a five-point Likert scale, where 4 represents an extremely strong influence, 3 is a strong influence, 2 represents an influence, 1 is a very small influence, and 0 implies no influence. The evaluation of links can be developed from interviews and workshops with stakeholders in projects. The Delphi method is adopted to minimize bias from dominant participants in the workshops. Ideally, all identified stakeholders should be involved in this step to achieve a consensus, but in reality, only some key stakeholders are engaged because of practical difficulties [24].

**Table 3:** An example of CSF-stakeholder evaluation matrix.

	CSF1	CSF2	CSF3	CSF4	CSF5
**S1**	0	3	1	2	3
**S2**	4	0	0	3	0
**S3**	0	4	3	2	1

### 3.3. Step 3: Visualization and Transformation of the Network

Based on the node and link identification, a network for CSFs and stakeholders in energy efficiency retrofit can be drawn. Different from the conventional one-mode network, this paper proposes a two-mode network to represent the relationship between CSFs and stakeholders.

**Figure 2:**
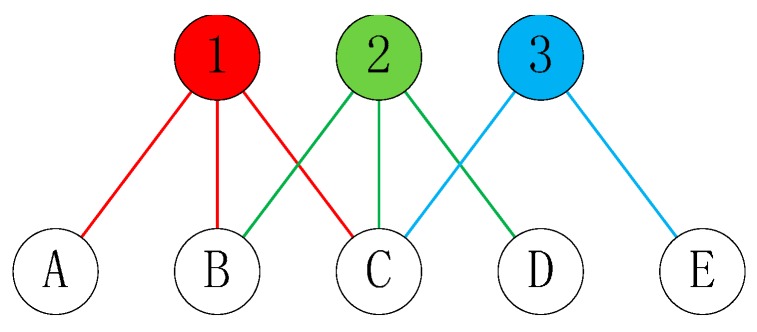
An example of two-mode network.

A two-mode network represents the relationship between the two groups. It can be represented as a triplet G = (▽, △, E), where ▽ is the set of top nodes, △ is the set of bottom nodes, and E ⊆▽ × △ is the set of links. Its difference with the classical one-mode network is that its nodes are in two disjoint sets, and the links are between a node of one set and another [22]. That is, two nodes in the same set should not be linked. Several large real-world networks may be modeled naturally by a bipartite graph when there are relationships between two different sets. For example, Breiger [49] analyzed personal relationships with a two-mode network by modeling the membership in different groups. Other real-world networks have been modeled by the two-mode network, such as the network of actors and movies, where actors are linked to movies in which they star [50]; firms and cities, where firms are linked to cities where they are located [51]; and authors and papers, where authors are linked to the published papers they wrote [52]. Figure 2 shows an example of a two-mode network.

Various methods and tools are used to analyze the classical one-mode network. However, there are only a few methods to analyze a two-mode network. The two-mode network has some specific notions, but most lack rigor and generality, making the results difficult to evaluate. Generally, a two-mode network should be converted into a one-mode network, which is then analyzed by classical methods. Although this conversion causes information loss, a significant amount of existing notions can be used for a one-mode network, and networks can be compared by unified methods. Therefore, conversion methods are widely used in two-mode network analysis.

**Figure 3:**
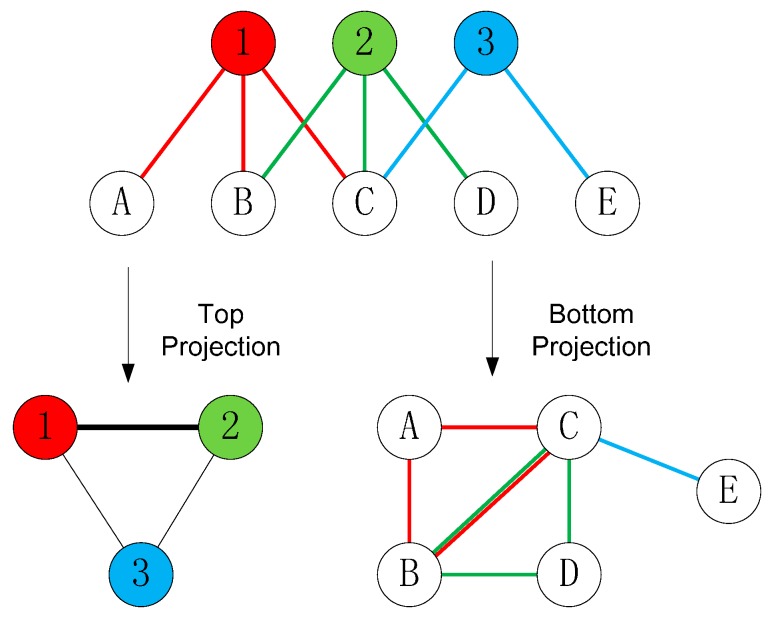
An example of top and bottom projection for two-mode network.

Projection is one of the most commonly used conversion methods, and can either be top or bottom projection. Top projection can be represented by G▽ = (▽, E▽), in which two nodes in ▽ are linked together if they are both linked to a common neighbor in △. The value of the link of G▽ can be defined as the number of common neighbors or weighed value of links in E. The bottom projection is processed by the same method as that for top projection. Figure 3 illustrates an example of top and bottom projection. Several previous studies used projection to transform and analyze a two-mode network. An actor-movie network can be projected to a one-mode network for actor relationship (*i.e.*, two actors are linked if they starred in the same movie) [50]. Similarly, a firm-city network can be projected to a network of firms that are linked together if they have branches in the same city [51].

This paper maps the interrelation between CSFs and stakeholders in energy efficiency retrofit on graph G, where ▽ represents stakeholders, △ represents CSFs, and E is the tightness degree between the stakeholder and CSF, which can be illustrated by weighted links on a scale of zero to four. To analyze CSFs, this two-mode CSF-stakeholder network is projected to a one-mode network for CSFs. This CSF network, which can be analyzed by classical one-mode analysis methods, shows the relationship of CSFs through stakeholders. The two-mode network can be visualized by various software packages, including UCINET, NetMiner, NetDraw, and Pajek [24].

### 3.4. Step 4: Network Analysis

After the visualization and projection of the two-mode network, the characteristics of CSFs can be determined by network and statistical analysis.

#### 3.4.1. Correlation Coefficient Analysis

Correlation coefficient analysis computes the similarity between two CSFs with the following equation:

(1) Coij=∑k=1n(eki−Ei¯)(ekj−Ej¯)[∑k=1n(eki−Ei¯)2(ekj−Ej¯)2]12where Ei¯=∑k=1nekin

$Co_{ij}$ is the correlation coefficient between $CSF_{i}$ and $CSF_{j}$; n is the number of stakeholders to which they are both related; $e_{ki}$ is weight of the edge, which links $CSF_{i}$ to the *k*th stakeholder ($S_{k}$); and $\bar{E_{i}}$ is the expectation of $e_{ki}$. $Co_{ij}$ is normalized to interval from −1 to 1. That is, the larger the value, the more similar are the CSFs; and the smaller the value, the more different are the CSFs.

The result of this correlation coefficient test indicates the similarity among CSFs according to their relationship with stakeholders. A high correlation coefficient value demonstrates that the two CSFs have a similar relationship with stakeholders. An extreme example is that the two CSFs obtain exactly the same points in every stakeholder, which means the similarity is one, and the two CSFs have the same relationship with stakeholders. Similar CSFs may have similar issues, benefits, risks, and effects on stakeholders. For example, in stakeholder management, project manager can categorize stakeholders related to similar CFSs in the same group. It can help stakeholders communicate with each other efficiently. For risk assessment and troubleshooting, when a CSF has problem, the most related CSFs can be quickly identified and controlled. Then, the related stakeholders can be gathered to address this problem. Therefore, this indicator can facilitate stakeholder management, risk assessment, troubleshooting and other project management aspects.

#### 3.4.2. Prioritization

Prioritization is an essential analysis of CSFs. It indicates the important CSFs that need to be emphasized and assigned high priority. To examine the priority rank of CSFs, centrality is used to show the status of every CSF in the network. The concept of centrality was developed by Freeman [53], and is widely used in network analysis. Degree and betweenness centrality are two of the most used indicators for centrality:

(1) Degree centrality is defined as the link that a node shares directly with other nodes, and is commonly used for the structural importance of nodes because it focuses on the local structure in which a particular node is embedded [47]. The node with a high degree centrality has direct weighted links to other nodes, which highly influences the neighbors.
(2) Betweenness centrality is the number of times a node is on the path between two not- interlinked nodes, and is commonly used for accessing the power of nodes [53]. It refers to the argument that a node links other nodes that were not directly linked previously [47]. The node with high betweenness centrality serves as a broker for neighbors because some neighbors will be apart without this node. Therefore, nodes with high betweenness centrality are considered to have considerable power over their neighbors.

Both degree and betweenness centrality are widely used in priority analysis in network [24,47,51]. Prell, Hubacek and Reed [48] indicated that degree and betweenness centrality have advantages and disadvantages. Therefore, the present study considers both and compares the results for a comprehensive analysis.

#### 3.4.3. Clustering

Different from classical clustering based on the score of every CSF, the present study clusters CSFs according to their relationship with stakeholders. The CSFs in the same cluster have a similar relationship with stakeholders. The result of this clustering method can help manage CSFs in practical projects. For example, CSFs in the same cluster can be assigned to a group of stakeholders that have frequent and tight interactions to improve the efficiency and effect of CSF management. There are several kinds of clustering techniques, such as a random, self-organizing feature map, hyper-graph partitioning, generalized k-means, and weighted graph partitioning [54].

### 3.5. Step 5: Presentation of Findings and Suggestions

Some innovative findings can be developed by comparing and mining the results of network analysis in the previous steps. Suggestions are provided based on the findings integrated with the state-of-the-art energy efficiency retrofit in China, including the CSFs prioritization, important stakeholders related on a particular CSF, and policies to improve CSF and stakeholder management.

## 4. Case Study

The Chinese government pays considerable attention to energy efficiency and carbon emission issues. It introduced policies to improve energy efficiency retrofit for existing buildings. In its 12th Five-year Plan (2011–2015), approximately 400 million m^2^ residential buildings and 60 million m^2^ public buildings are planned to be retrofitted in 2011–2015 as pilot projects, and more energy efficiency retrofit projects are planned to be implemented in the future.

Based on these energy efficiency retrofit practices, a case study with the proposed method was conducted to reveal the stakeholder-associated CSFs for energy efficiency retrofits in China. The case study illustrates how the proposed method works stepwise. To build a two-mode network in energy efficiency retrofit, CSFs and stakeholders were identified through a literature review. In-depth interviews were used to refine the list. Then, a workshop was conducted to evaluate the strength of the relationship between CSFs and stakeholders. Finally, a two-mode network was built based on the results of the workshop.

### 4.1. Building the Two-Mode Network

From the literature review, a series of semi-structured interviews was conducted with 16 experts who have been involved in energy efficiency retrofit projects. Ten of these experts were involved in the energy efficiency retrofit project for the building of the headquarters of China Resource in Hong Kong. These experts were the project director, technical specialist, and purchasing specialist. The other six experts were from Mainland China and they participated in energy efficiency retrofit projects as designer, researcher, contract manager, contractor, or third-party institute authorized by the government to audit projects. The interviews focused on the following issues: (1) CSFs in energy efficiency retrofit projects, (2) main stakeholders related to project success, and (3) categories of CSFs and categorization of factors. The interviews lasted from 30 min to 2 h in March to July 2014. The lists of CSFs and stakeholders were refined based on the qualitative analysis of interview data, which is summarized in Table 1 and Table 2. This study focuses only on “green” CSFs and stakeholders.

A workshop with classical experience-based methods was held to evaluate the degree of tightness between the stakeholder and CSF, namely, the links in the network. The Delphi method was used to collect independent opinions from the professionals with two rounds of modification. An open discussion was conducted among the professionals to reach a consensus. A CSF-stakeholder matrix with 28 CSFs and 13 stakeholders was developed based on the data from the workshop, as shown in Table S1. The two-mode network was translated from the CSF-stakeholder matrix with the SNA software “netminer” (CYRAM Company, Seoul, Korea). Figure S1 shows the two-mode network, where the shape of the nodes indicates stakeholders and the different types of CSFs.

### 4.2. Result of the Network Analysis

#### 4.2.1. Correlation Coefficient of CSFs

**Figure 4:**
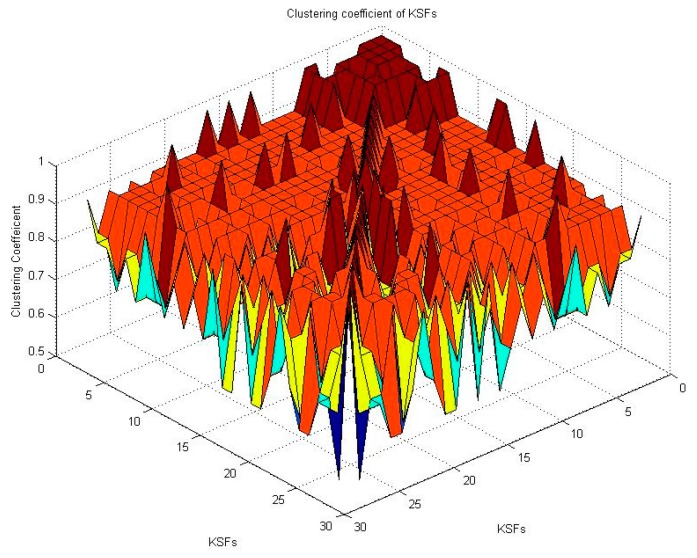
Correlation coefficient of CSFs in 3D model.

The correlation coefficient of CSFs is calculated by Equation (1). Figure 4 shows the correlation coefficient of CSFs, where the color closer to red implies a higher correlation coefficient, and that closer to blue implies a lower correlation coefficient. The order of CSFs is re-arranged according to the value of correlation coefficient, shown in Table S2. High scores of correlation coefficient (*e.g.*, CSF24 has the highest correlation coefficient with CSF 22, 26, 17, 25, and 23) indicate that related factors are similar in a social network from the stakeholder perspective.

#### 4.2.2. Prioritization of CSFs

**Figure 5:**
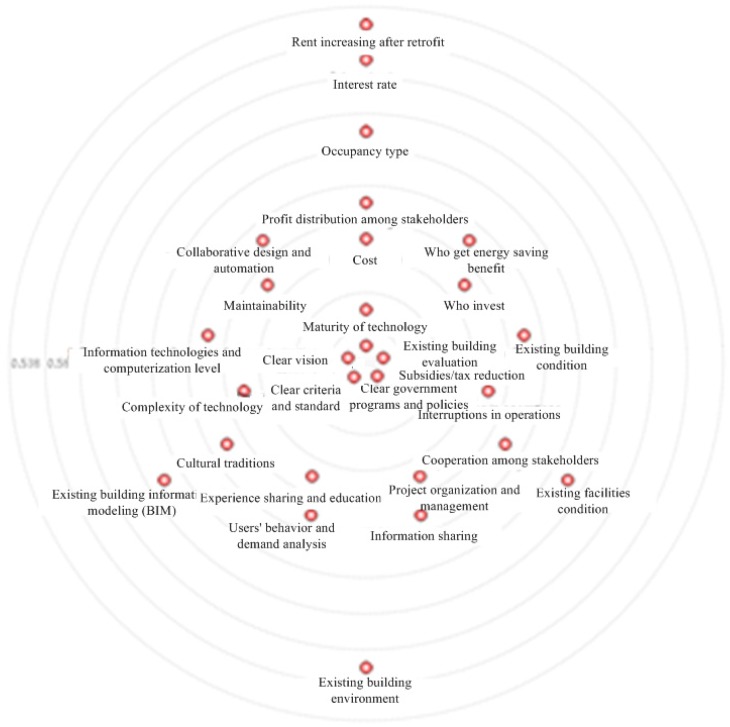
Degree centrality of CSFs.

**Figure 6:**
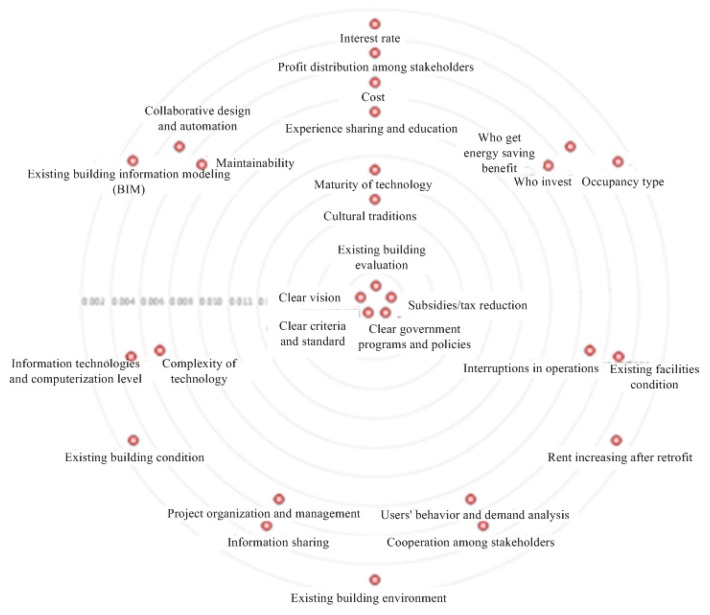
The betweenness centrality of CSFs.

The importance of CSFs is represented by both degree and betweenness centrality, as mentioned in Section 3.4. The degree and betweenness centrality are shown in Figure 5 and Figure 6, and the importance ranks of CSFs based on them are shown in Table 4.

The ranks based on degree and betweenness centrality are different, but the five most important factors are the same. These factors are clear criteria and standard, clear government programs and policies, clear vision, existing building evaluation, and subsidies or tax reduction. Three of these CSFs; namely, clear criteria and standard, clear government programs and policies, and subsidies or tax reduction; are directly related to the government, thus indicating that the government plays an important role in energy efficiency retrofit projects in China. The other two CSFs belong to the sociocultural and building-information categories. Stakeholders, especially clients, are considered sensitive to economic benefits [10], but some CSFs that are directly related to benefits, such as “rent increasing after retrofit” and “interest rate”, are ranked lower than stakeholders. One reason of this result is that the centrality rank indicates the relation strength of a CSF with others. A CSF related to fewer CSFs through stakeholders should have lower rank. For example, “rent increasing after retrofit” is very important to owners and occupiers. But other stakeholders (*e.g.*, contractor and supplier), do not value this factor. Namely, this factor does not have strong relationship with others. Therefore, this factor ranks last. Another reason of this result might be that these factors do not affect the project success significantly in short time. For example, the “interest rate” is important to payback time, but the payback time of energy efficiency retrofit projects is relatively long [55]. Therefore, the “interest rate” is in higher uncertainty and has lower rank.

**Table 4:** Priority rank of CSFs based on degree and betweenness centrality.

CSF		Degree Centrality		Betweenness Centrality	
		Value	Rank	Value	Rank
CSF28	Clear criteria and standard	1	1	0.02085	1
CSF27	Clear government programs and policies	1	1	0.02085	1
CSF15	Clear vision	1	1	0.02085	1
CSF14	Existing building evaluation	1	1	0.02085	1
CSF8	Subsidies/tax reduction	1	1	0.02085	1
CSF22	Maturity of technology	0.92	6	0.01169	7
CSF24	Maintainability	0.85	7	0.00618	10
CSF23	Complexity of technology	0.85	7	0.00618	10
CSF20	Experience sharing and education	0.85	7	0.00927	8
CSF19	Project organization and management	0.85	7	0.00618	10
CSF4	Interruptions in operations	0.85	7	0.00618	10
CSF2	Who invest	0.85	7	0.00618	10
CSF1	Cost	0.85	7	0.00618	10
CSF26	Collaborative design and automation	0.77	14	0.00427	20
CSF25	Information technologies and computerization level	0.77	14	0.00427	20
CSF21	Cultural traditions	0.77	14	0.01341	6
CSF18	Users' behavior and demand analysis	0.77	14	0.00726	9
CSF17	Information sharing	0.77	14	0.0043	16
CSF16	Cooperation among stakeholders	0.77	14	0.0043	16
CSF11	Existing building condition	0.77	14	0.00427	20
CSF7	Who get energy saving benefit	0.77	14	0.0043	16
CSF3	Profit distribution among stakeholders	0.77	14	0.0043	16
CSF13	Existing building information modeling (BIM)	0.69	23	0.00347	23
CSF12	Existing facilities condition	0.69	23	0.00263	25
CSF6	Occupancy type	0.69	23	0.00263	25
CSF10	Existing building environment	0.62	26	0.00193	28
CSF5	Interest rate	0.62	26	0.00272	24
CSF9	Rent increasing after retrofit	0.54	28	0.00202	27

#### 4.2.3. Clusters of CSFs

Clustering analysis is completed by using Ward’s method [56]. Four clusters are listed in Table 5. Cluster 1 is the building condition cluster, which is related to the existing and future conditions during retrofit, such as building environment and interruptions in operations. Cluster 2 is the benefit cluster, in which factors are about the benefits that stakeholders can gain from retrofit, including profit distribution among stakeholders and rent increase after retrofit. Cluster 3 is the construction cluster, which involves factors related to the construction process, including evaluation, design, construction, and operation phases. Cluster 4 is the policy and investment cluster, which is tightly linked to the government and clients. The factors in Cluster 4 are essential in making decisions on whether to undergo retrofit.

**Table 5:** Four clusters of CSFs.

Number	Cluster 1	Cluster 2	Cluster 3	Cluster 4
1	CSF13	CSF5	CSF23	CSF27
2	CSF12	CSF9	CSF22	CSF28
3	CSF11	CSF7	CSF20	CSF8
4	CSF10	CSF3	CSF26	CSF19
5	CSF6		CSF25	CSF16
6	CSF4		CSF24	CSF1
7			CSF21	CSF2
8			CSF18	
9			CSF15	
10			CSF14	
11			CSF17	

## 5. Discussion and Conclusions

This study proposes an innovative social network-based method to analyze CSFs associated with stakeholders in energy efficiency retrofit projects. The analysis result based on the proposed method could reveal the characteristics of CSFs through the relationship with stakeholders. Different from the classical one-mode network in previous studies, the two-mode network is adopted to model CSFs and stakeholders as two node groups, with the degree of tightness between CSFs and stakeholders as links in the network. To apply this theoretically innovative model, a structured procedure is provided to indicate how to identify nodes and links, build the network, and analyze it stepwise. This method can improve the effectiveness and accuracy of CSF analysis by supplementing stakeholder influence in the traditional CSF analysis. The analysis results can facilitate CSF identification and project management in energy efficiency retrofit.

A case study was conducted to analyze the CSFs for energy efficiency retrofit projects with the proposed method. The following important findings are obtained: (1) Similarity between two CSFs is measured by the correlation coefficient to show which factors have a similar relationship with stakeholders. (2) Although the priority rank based on degree centrality is different from that based on betweenness centrality, the five most important factors, namely, clear criteria and standard, clear government programs, clear vision, existing building evaluation and policies, and subsidies or tax reduction, are the same. (3) Factors related to the government and policy are significantly important in energy efficiency retrofit projects in China, unlike in other countries (*e.g.*, Yang and Zou [24] indicated that the government does not play an important role in green building development in Australia). (4) Unlike the research that focused on the technical factors of retrofit [26], factors related to sociocultural and policies are more emphasized by project practitioners. (5) CSFs are divided into four clusters; namely, building condition, benefit, construction, and policy and investment clusters. The results are based on the opinions of several professionals and need to be verified through further studies. The findings of this case study are meaningful and expected to help decision-makers involved in green building projects.

The study has four research limitations. First, the stakeholders identified through the literature review and interviews were all internal stakeholders involved in previous energy efficiency retrofit projects. External stakeholders, such as the media, were excluded. Second, this case study used classical experience-based methods to identify CSFs and stakeholders for practical reasons. Therefore, the number of experts was limited. Although snowball sampling is time consuming and practically challenging, it is considered better than other methods in identifying a complete and accurate list. Third, the workshop was a one-off. For general results, the proposed method should be applied in more workshops so that results could be reviewed and compared to improve the understanding of CSFs for energy efficiency retrofit. Fourth, this case study only analyzes stakeholders statically. In particular, it does not differentiate stakeholders and their influence in different phases.

Further research can focus on some potential areas and directions. Future studies can use other analysis methods besides projection, although the methods for a two-mode network are limited. These methods have advantages and disadvantages. Therefore, other two-mode network methods can be adopted to facilitate the CSF-stakeholder network analysis. Second, aside from CSFs, other aspects of project management can be studied by this two-mode network method, such as risk-stakeholder, incentive-stakeholder, and CSFs-process networks. Third, the proposed method can be further applied in dynamic analysis. Different relationships among stakeholders and CSFs in different phases can be investigated and compared. Finally, more case studies should be conducted to obtain more concrete findings. The analysis results can be compared with other relevant results, such as CSFs for energy efficiency retrofit in other countries, and for new green building construction projects. The comparative results can reveal the particular characteristics of CSFs for energy efficiency retrofit in different backgrounds.

This study contributes to CSF analysis by developing an innovative CSF-stakeholder two-mode network method, which serves as a basis for future research in SNA-based CSF evaluation. The CSFs in this study were identified within the context of energy efficiency retrofits in China. Therefore, they cannot be simply applied to other countries or project types. However, the research method and analytical model can be applied to other research areas.

## Supplementary Files

Supplementary File 1
