# Supplementary material for: Improving Management of Green Retrofits from a Stakeholder Perspective: A Case Study in China
Source: Int J Environ Res Public Health. 2015 Oct 28;12(11):13823–42. doi: 10.3390/ijerph121113823 (PMC4661617; doi:10.3390/ijerph121113823)
Supplement: Supplementary File 1 [file ijerph-12-13823-s001.pdf]

Supplementary Information

Improving Management of Green Retrofits from a Stakeholder Perspective: A Case Study in China

Table S1. CSF-stakeholder matrix based on data from the workshop.

	S1	S2	S3	S4	S5	S6	S7	S8	S9	S10	S11	S12	S13
**CSF1**	4	2	2	3	2	2	2	2	2	3	0	0	1
**CSF2**	4	1	2	2	2	2	2	3	2	4	0	0	1
**CSF3**	4	2	3	1	1	1	1	2	1	2	0	0	0
**CSF4**	3	4	4	2	2	1	2	2	1	1	0	0	1
**CSF5**	3	0	1	1	0	1	1	2	3	2	0	0	0
**CSF6**	3	3	3	2	2	1	1	1	0	1	0	0	0
**CSF7**	4	3	3	1	1	1	1	2	1	2	0	0	0
**CSF8**	4	2	2	1	1	1	1	4	2	2	1	1	1
**CSF9**	4	4	3	1	0	0	0	1	2	1	0	0	0
**CSF10**	3	3	3	2	1	1	0	1	0	1	0	0	0
**CSF11**	3	3	2	3	1	1	1	1	0	1	0	0	1
**CSF12**	3	3	3	3	1	2	2	1	0	2	0	0	0
**CSF13**	3	2	3	3	2	2	2	1	0	0	0	0	1
**CSF14**	3	2	3	2	1	2	1	2	1	2	1	1	2
**CSF15**	3	2	2	3	1	1	1	3	1	1	1	1	2
**CSF16**	3	1	2	2	2	2	2	3	2	3	0	0	0
**CSF17**	3	2	3	3	1	1	2	3	1	3	0	0	0
**CSF18**	3	3	3	3	1	1	1	3	0	2	1	0	0
**CSF19**	3	2	2	2	3	2	3	2	1	2	0	0	1
**CSF20**	3	1	2	4	2	1	1	3	0	2	1	0	2
**CSF21**	3	2	3	3	0	1	0	3	0	2	1	1	2
**CSF22**	4	1	3	4	2	2	1	3	1	3	1	0	1
**CSF23**	4	1	3	4	3	2	2	3	1	2	0	0	1
**CSF24**	4	2	4	3	2	2	2	3	1	2	0	0	1
**CSF25**	3	2	3	3	2	1	1	2	0	3	0	0	1
**CSF26**	4	2	3	3	2	1	1	2	0	2	0	0	1
**CSF27**	4	2	2	3	2	2	2	4	2	3	1	1	1
**CSF28**	3	1	2	4	3	3	3	4	1	3	1	1	2

Table S2. Correlation coefficient matrix of CSFs.

Number of CSFs	1	24	22	26	17	25	27	23	14	3	18	7	19	16	11	6	2	12	4	15	8	20	10	13	21	28	9	5
**1**	1	1	1	0.9	0.9	0.9	1	1	0.9	0.9	0.9	0.9	1	1	0.9	0.9	1	0.9	0.9	0.9	0.9	0.9	0.8	0.9	0.8	0.9	0.8	0.9
**24**		1	1	1	1	1	0.9	1	0.9	0.9	0.9	0.9	0.9	0.9	0.9	0.9	0.9	0.9	0.9	0.9	0.9	0.9	0.9	0.9	0.9	0.9	0.8	0.8
**22**			1	1	0.9	1	1	1	0.9	0.9	0.9	0.9	0.9	0.9	0.9	0.8	0.9	0.9	0.8	0.9	0.9	1	0.8	0.9	0.9	0.9	0.7	0.8
**26**				1	0.9	1	0.9	1	0.9	0.9	0.9	0.9	0.9	0.9	0.9	0.9	0.9	0.9	0.9	0.9	0.9	0.9	0.9	0.9	0.9	0.9	0.8	0.7
**17**					1	1	0.9	0.9	0.9	0.9	1	0.9	0.9	0.9	0.9	0.9	0.9	0.9	0.9	0.9	0.9	0.9	0.9	0.8	0.9	0.9	0.8	0.8
**25**						1	0.9	0.9	0.9	0.9	0.9	0.9	0.9	0.9	0.9	0.9	0.9	0.9	0.9	0.9	0.8	0.9	0.9	0.9	0.9	0.9	0.7	0.7
**27**							1	0.9	0.9	0.9	0.9	0.9	0.9	1	0.8	0.8	1	0.9	0.8	0.9	1	0.9	0.8	0.8	0.9	1	0.7	0.9
**23**								1	0.9	0.9	0.9	0.8	0.9	0.9	0.9	0.9	0.9	0.9	0.9	0.9	0.8	0.9	0.8	0.9	0.8	0.9	0.7	0.8
**14**									1	0.9	0.9	0.9	0.9	0.9	0.9	0.9	0.9	0.9	0.9	0.9	0.9	0.9	0.9	0.9	0.9	0.9	0.8	0.7
**3**										1	0.9	1	0.9	0.9	0.9	0.9	0.9	0.9	0.9	0.8	0.9	0.8	0.9	0.8	0.8	0.8	0.9	0.8
**18**											1	0.9	0.8	0.9	0.9	0.9	0.8	0.9	0.9	0.9	0.9	0.9	0.9	0.9	0.9	0.8	0.8	0.7
**7**												1	0.9	0.9	0.9	0.9	0.9	0.9	0.9	0.8	0.9	0.8	0.9	0.8	0.8	0.8	0.9	0.8
**19**													1	0.9	0.9	0.9	0.9	0.9	0.9	0.9	0.8	0.9	0.8	0.9	0.8	0.9	0.7	0.7
**16**														1	0.8	0.8	1	0.8	0.8	0.8	0.9	0.8	0.8	0.8	0.8	0.9	0.7	0.9
**11**															1	0.9	0.8	1	0.9	0.9	0.8	0.9	0.9	0.9	0.9	0.8	0.8	0.6
**6**																1	0.8	1	1	0.8	0.8	0.8	1	0.9	0.8	0.8	0.9	0.6
**2**																	1	0.8	0.8	0.8	0.9	0.9	0.7	0.8	0.8	0.9	0.7	0.9
**12**																		1	0.9	0.8	0.8	0.8	0.9	0.9	0.8	0.8	0.8	0.6
**4**																			1	0.9	0.8	0.8	0.9	0.9	0.8	0.8	0.9	0.6
**15**																				1	0.9	0.9	0.8	0.9	0.9	0.9	0.7	0.7
**8**																					1	0.8	0.8	0.7	0.9	0.8	0.8	0.9
**20**																						1	0.8	0.9	0.9	0.9	0.6	0.7
**10**																							1	0.9	0.9	0.7	0.9	0.6
**13**																								1	0.8	0.8	0.7	0.6

Table S2. *Cont.*

Number of CSFs	1	24	22	26	17	25	27	23	14	3	18	7	19	16	11	6	2	12	4	15	8	20	10	13	21	28	9	5
**21**																									1	0.8	0.7	0.7
**28**																										1	0.5	0.7
**9**																											1	0.7
**5**																												1

Note: The order of CSFs is re-arranged according to the value of correlation coefficient.

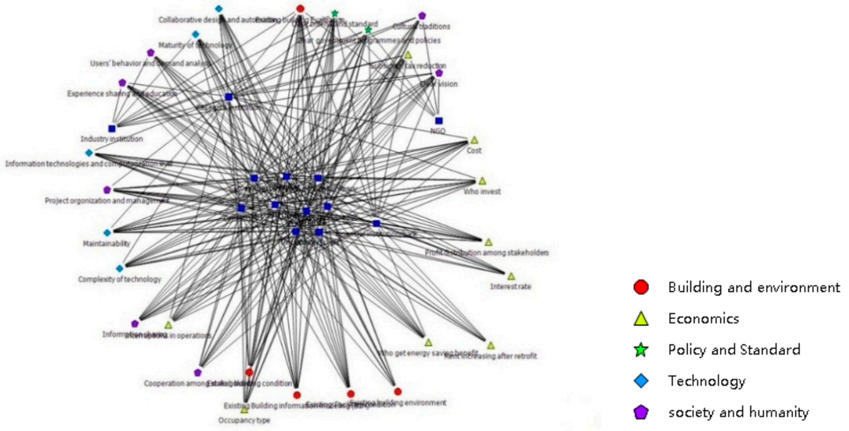

Figure S1. Two-mode network of CSFs and stakeholders in energy efficiency retrofit.

© 2015 by the authors; licensee MDPI, Basel, Switzerland. This article is an open access article distributed under the terms and conditions of the Creative Commons Attribution license (http://creativecommons.org/licenses/by/4.0/).
